# Tools and instruments for needs assessment, monitoring and evaluation of health research capacity development activities at the individual and organizational level: a systematic review

**DOI:** 10.1186/s12961-015-0070-3

**Published:** 2015-12-21

**Authors:** Johanna Huber, Sushil Nepal, Daniel Bauer, Insa Wessels, Martin R Fischer, Claudia Kiessling

**Affiliations:** Institut für Didaktik und Ausbildungsforschung in der Medizin, Klinikum der Universität München, Ziemssenstraße 1, 80336 Munich, Germany; bologna.lab, Humboldt-Universität zu Berlin, Hausvogteiplatz 5-7, 10117 Berlin, Germany; Medizinische Hochschule Brandenburg Theodor Fontane, Fehrbelliner Straße 38, 16816 Neuruppin, Germany

**Keywords:** Health research capacity development, Individual level, Monitoring and evaluation, Needs assessment, Organizational level, Tools

## Abstract

**Background:**

In the past decades, various frameworks, methods, indicators, and tools have been developed to assess the needs as well as to monitor and evaluate (needs assessment, monitoring and evaluation; “NaME”) health research capacity development (HRCD) activities. This systematic review gives an overview on NaME activities at the individual and organizational level in the past 10 years with a specific focus on methods, tools and instruments. Insight from this review might support researchers and stakeholders in systemizing future efforts in the HRCD field.

**Methods:**

A systematic literature search was conducted in PubMed and Google Scholar. Additionally, the personal bibliographies of the authors were scanned. Two researchers independently reviewed the identified abstracts for inclusion according to previously defined eligibility criteria. The included articles were analysed with a focus on both different HRCD activities as well as NaME efforts.

**Results:**

Initially, the search revealed 700 records in PubMed, two additional records in Google Scholar, and 10 abstracts from the personal bibliographies of the authors. Finally, 42 studies were included and analysed in depth. Findings show that the NaME efforts in the field of HRCD are as complex and manifold as the concept of HRCD itself. NaME is predominately focused on outcome evaluation and mainly refers to the individual and team levels.

**Conclusion:**

A substantial need for a coherent and transparent taxonomy of HRCD activities to maximize the benefits of future studies in the field was identified. A coherent overview of the tools used to monitor and evaluate HRCD activities is provided to inform further research in the field.

## Background

The capacity to cope with new and ill-structured situations is a crucial ability in today’s world. Developing this ability, by shaping empowered citizens, challenges individuals as well as organisations and societies. This process of empowerment is usually referred to as capacity development (CD) [1]. While this term has been commonly used for years in the field of foreign aid, other societal and political domains (e.g. social work, education and health systems) are increasingly adopting the concept of CD when developing new or existing competencies, structures, and strategies for building resilient individuals and organizations [2]. Also in the field of health research, an increasing number of activities to strengthen health research competencies and to support organizations can be observed – as demanded by the three United Nations Millennium Development Goals addressing health related issues [3–6]. Several frameworks are already in use that support a structured approach to health research capacity development (HRCD) and address competencies that are specific to health research [7–9]. These frameworks usually incorporate the individual or team, organization or institution, and society levels [8, 10, 11]. One conclusion that can be drawn from the available evidence is that, in such a structured approach to HRCD efforts, meaningful data collection is crucial. First, data collection incorporates the HRCD needs assessment and second, the monitoring and evaluation (NaME) of activities and programs once implemented. Therefore, HRCD activities should address the needs as assessed. Monitoring and evaluation of these activities should reflect the desired outcomes as defined beforehand [12–15]. Bates et al. [16] indicate how data collection tools and instruments are usually developed for a certain purpose in a certain context. The context specificity of tools and instruments has to be considered and the appropriateness of these must be determined when selecting instruments for any needs assessment for a new project. This article offers a systematic review of tools and instruments for the NaME of HRCD activities at the individual or team and the organizational levels to aid HRCD initiatives in selecting appropriate tools and instruments for data collection within their respective context. For this purpose, a range of studies published between January 1, 2003, and June 30, 2013, were chosen and analysed based on different context parameters such as the level of the CD and the nature of the HRCD activities.

## Methods

We followed the PRISMA checklist for reporting systematic reviews and meta-analyses [17]. Inclusion and analysis criteria were defined in advance and documented in a protocol (Tables 1 and 2).

**Table 1:** **Description and operationalization of the five inclusion categories**

Category	Description/Operationalization
Capacity development	“Capacity development is the process through which people, organizations and society shape their own development and adapt it to changing conditions and frameworks” [18]
Research	Research spider [19]: - writing a research protocol - using qualitative research methods - publishing research - writing and presenting a research report - analysing and interpreting results - using quantitative research methods - critically reviewing the literature - finding relevant literature - generating research ideas - applying for research funding Additional aspects developed according to [20] - leading teams - coordinating a research project - assuring the quality of work - considering ethical aspects in research
Health profession fields	Medicine, pharmacy, nursing, physical therapy, and other allied health professions
Monitoring and evaluation	- defining requirements - analysing current state - defining needs - assessing short- and mid-term outcomes - measuring long-term impact See also Figure 1
Level of NaME	- individual/team capacities to conduct research according to the operationalization of ‘research’ - organisational [10] aspects defined according to [18] ○ management and leadership ○ mission, vision, plan ○ human resources ○ culture ○ structures, processes and results

**Table 2:** **Nine aspects for further analysis of the included studies**

Aspect	Explanation
Authors’ name and year of publication	–
Country or region	… where the HRCD activity was conducted or the participants originated from; additionally classified according to the World Banks classification in low-, lower-middle, upper-middle and high-income economies; if disclosed in article
Study participants or material analysed	Study participants are people, who received the health research capacity development activity and were part of the needs assessment and monitoring and evaluation (NaME) study; additional, sample size and professional background of participants is given; or number and description of material analysed; if disclosed in article
Objective(s) of the study	See Table 3
capacity development activity	If applicable
Study design	Study designs were differentiated between single study approaches (e.g. an intervention study) and multi-study approaches (e.g. a combination of an intervention study with a non-intervention study); see also Figure 2
Level of NaME	Individual/team and/or organizational level
Focus of NaME	According to NaME framework; see Table 1 and Figure 1
Tools and instruments used for NaME	Additional information on mode of analysis (quantitative, qualitative, or mixed)

### Information sources and search strategy

We conducted the systematic literature search in July 2013. The search was done in both the literature database PubMed and the search engine Google Scholar. We applied the three search terms “capacity building” AND “research”, “capacity development” AND “research”, and “capacity strengthening” AND “research”. We checked the first 200 hits in Google Scholar for each search term. “Health” and “evaluation” were not included in the search terms as a pre-test search had revealed this would exclude relevant literature. Articles from personal bibliographies of the authors were also included.

### Inclusion categories and criteria

The inclusion process was structured along the five inclusion categories ‘capacity development’, ‘research’, ‘health profession fields’, ‘monitoring and evaluation’, and ‘level of NaME’. Table 1 gives a detailed overview of all descriptions and operationalisations used.

**Fig. 1:**
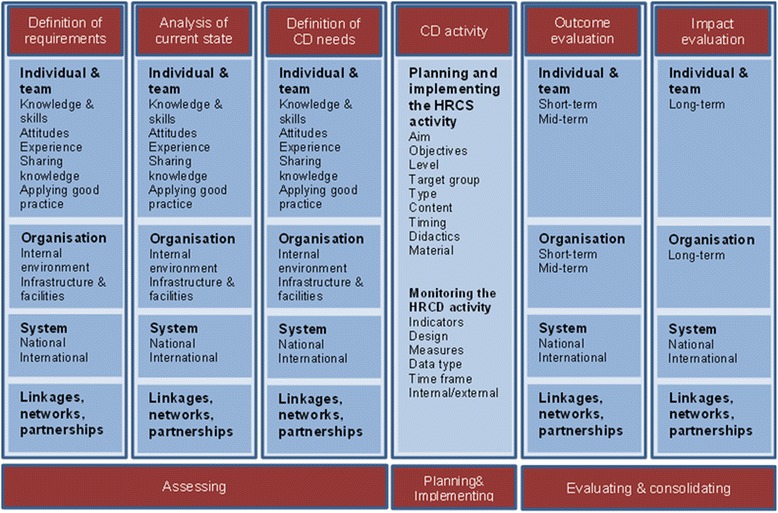
**Framework for needs assessment, monitoring and evaluation (NaME) of health research capacity development (HRCD) [** 2 **,** 5 **,** 8 **,** 10 **–** 13 **,** 15 **,** 21 **–** 25 **].**

The category ‘capacity development’ [18] represents an exemplary definition which serves as a guideline for inclusion but should not to be applied word by word. ‘Research’ was operationalized according to the categories of the ‘research spider’ [19]. Some process-related research skills as well as communicational and interpersonal skills were added to our operationalisation [20]. Main health professions were identified and grouped within different fields. NaME was operationalized according to a self-constructed NaME framework of HRCD activities (Fig. 1), which summarizes 13 HRCD/NaME frameworks [2, 5, 8, 10–13, 15, 21–25] and reflects the level of HRCD, common indicators, and the order (from needs assessment to impact evaluation) commonly used in the original frameworks.

For the categories ‘research’, ‘health profession fields’ and ‘monitoring and evaluation’, at least one of the operationalisations of each category had to be addressed by the study. The category ‘level of NaME’ was operationalized referring to the ESSENCE framework ‘Planning, monitoring and evaluation framework for capacity strengthening in health research’ which describes three CD levels: individual and/or team, organizational, and system levels [10]. Only publications focussing on NaME on the individual/team and organizational levels were considered for this review.

**Fig. 2:**
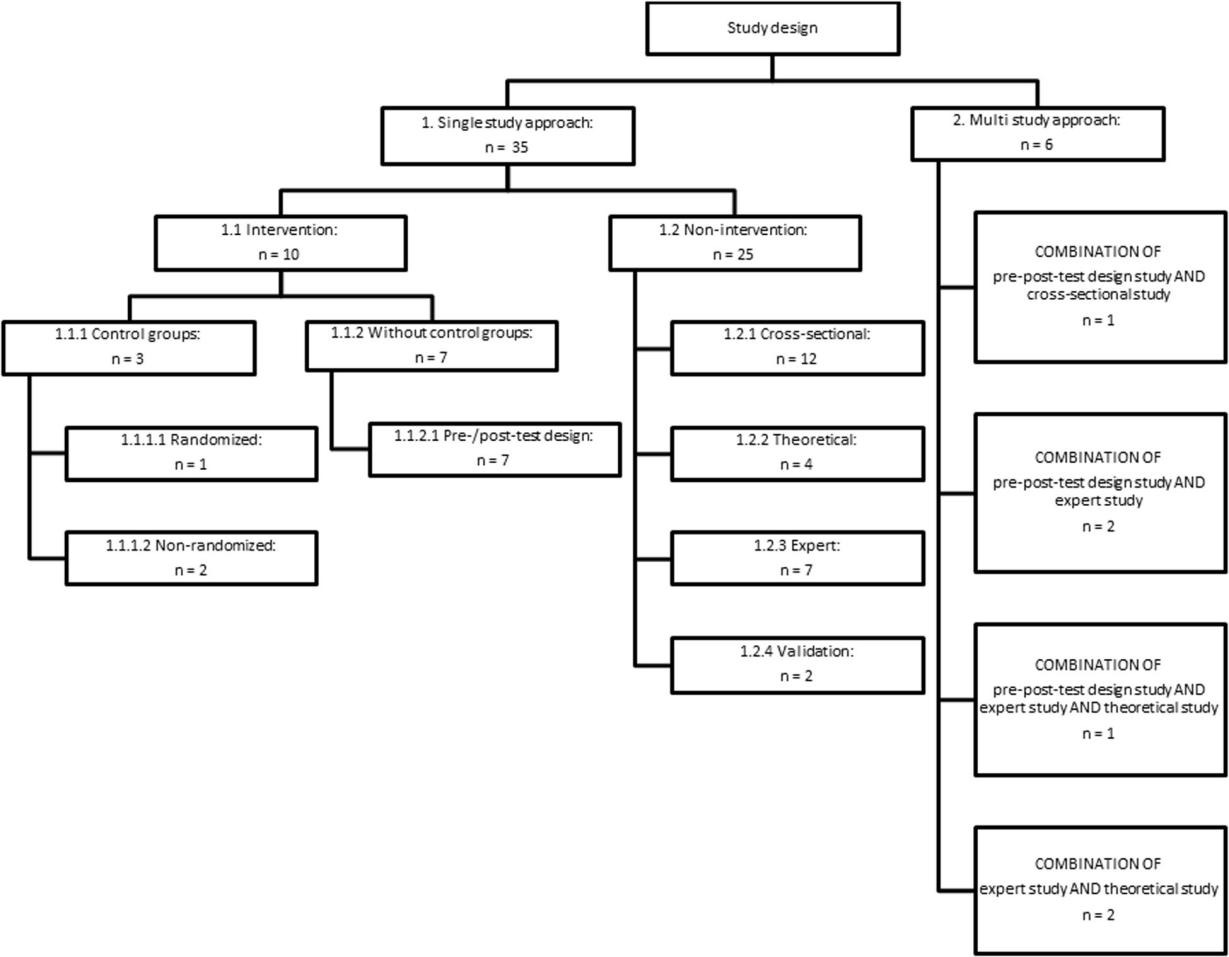
**Categorization of the study designs.** The study designs are restricted to the included studies.

Additionally, the following eligibility criteria were set: English or German language, publication period from January 1, 2003, to June 30, 2013, intervention, non-intervention and multiple design studies (Fig. 2). We excluded grey literature, editorials, comments, congress abstracts, letters, and similar. Articles focussing on institutional networks with external partners were excluded as well.

### Study selection

Two researchers, JH and SN, independently scanned the abstracts identified for inclusion. In case of disagreement, JH and SN discussed the abstracts in question. If consensus could still not be reached, a third reviewer, CK, was consulted. After consensus on inclusion was reached, the full-texts of all included studies were rechecked for inclusion by JH and SN.

### Study analysis procedure

We analysed the included articles according to nine aspects defined in Table 2.

## Results

**Fig. 3:**
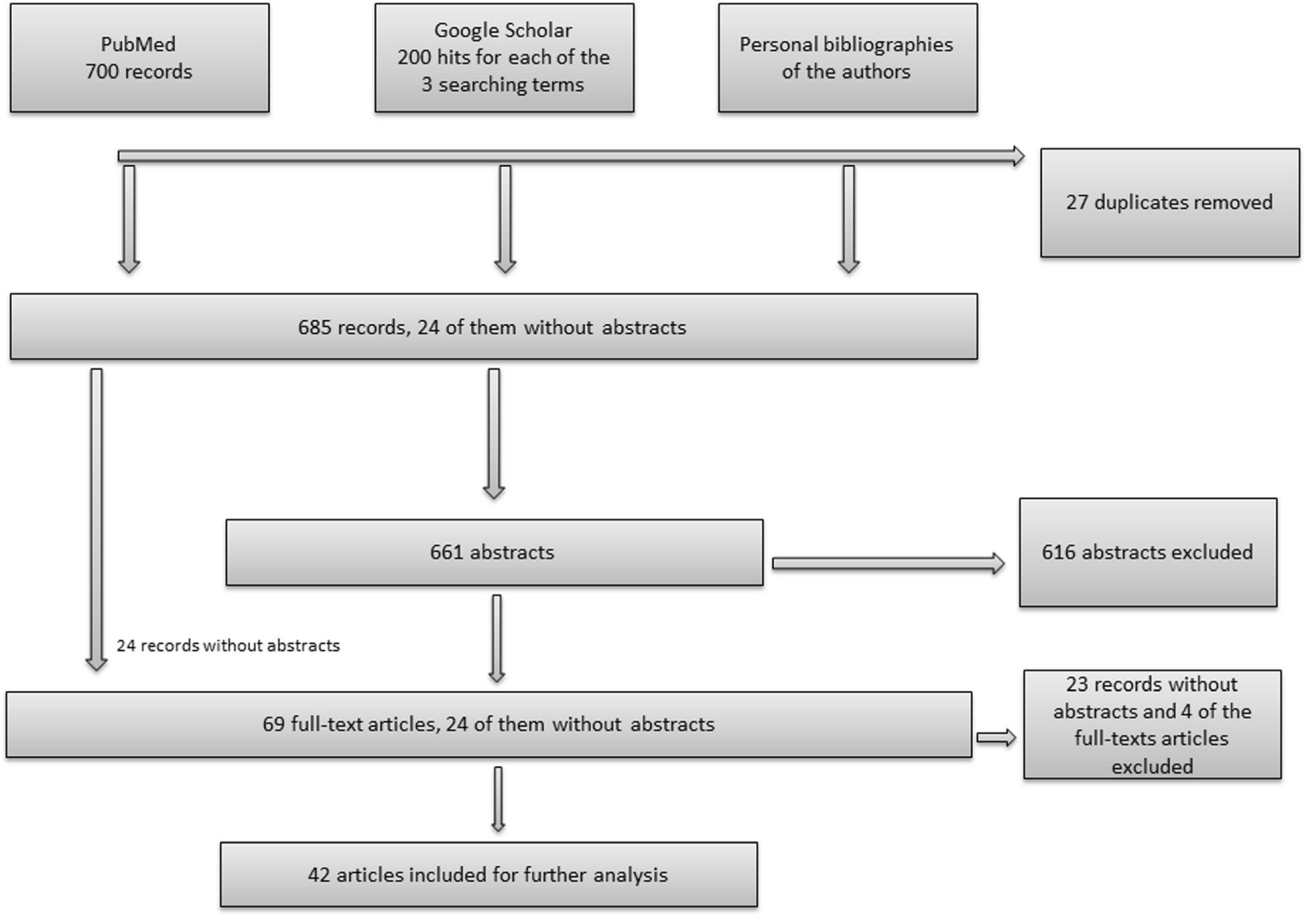
**Flowchart of the inclusion process.**

The search in PubMed revealed 700 suitable records (Fig. 3). We removed 27 duplicates, resulting in 673 records for inclusion screening. The first 200 hits for each of the three search terms in Google Scholar were considered, resulting in two additional records after removing duplicates. Furthermore, we included articles from the personal bibliographies of the authors, adding 10 more abstracts after checking for duplicates. Of the 685 records identified, 24 did not contain an abstract, but were preliminarily included for the full-text screening. JH and SN scanned the remaining 661 abstracts in terms of the inclusion criteria, thus excluding 616 records; 45 abstracts and the 24 records without abstracts were considered for full-text screening. After the full-text screening, 42 articles were finally included for further analysis; 37 articles originated from PubMed, one from Google Scholar, and four from the personal bibliographies of the authors.

These 42 articles were subsequently analysed along nine aspects (Table 2). The results are summarized in Table 3.

**Table 3:** **Included studies on needs assessment, monitoring and evaluation (NaME) of health research capacity development (HRCD) at the individual and organizational level**

No.	First author and year	Country/Region (country group)^a^	Participants (n^b^)/ Analysed material	Objective(s) of the study	Capacity development activity	Study design^c^	Level of NaME	Focus of NaME	Tools and instruments used for NaME (mode of analysis)
1	Ajuwon [34]	Nigeria (LMIC)	Physicians, dentists, nurses, laboratory scientists, and public health professionals of 29 governmental and two non-governmental organizations^d^	To evaluate training on research ethics	Workshop	2. Multi-study approach: expert study AND Intervention study in pre-post-test design	Individual	Definition of needs: quality of ethics review, good ethical consideration, planning and implementation of ethics training Outcome evaluation: knowledge and ethical reasoning	Focus group discussions and in-depth interviews for needs assessment (qualitative); 23-item-questionnaire for pre- and post-course evaluation (quantitative)
2	Ali [43]	13 African countries^e^	Health professionals, ethics committee members, scholars, journalists and scientists (n = 28)	To evaluate the Johns Hopkins-Fogarty African Bioethics Training Programme (FABTP)	One-year non-degree training	1.2.1 Cross-sectional study	Individual	Outcome evaluation: grants, publications, participants’ teaching activities	FABTP evaluation framework: Individual development (qualitative); Programme evaluation (quantitative)
3	Barchi [44]	Botswana (UMIC)	University faculty members^e^, community and governmental staff, research staff from non-governmental organisations, students (n = 71)	To evaluate training on research ethics	One-semester training programme	1.1.1.1 Intervention study in randomized controlled design	Individual	Outcome evaluation: knowledge and critical reasoning	Pre- and post-training delivery of Family Health International 40-item-test (quantitative); Self-constructed post-training case work with ethical challenges (quantitative)
4	Bates [21]	Ghana (LMIC)	Clinicians, physiotherapists and hospital managers^d^	To develop an evidence-based tool to guide the design, implementation, and evaluation of health research capacity development programmes	Not described further	1.2.2 Theoretical study	Individual and organizational	Mapping of the developed evaluation tool to identify needs and gaps: role of partners, institutional research support services, diplomas, research scope, educational quality assurance, publications, grants, use and dissemination of research within and outside of the organization	Validation of proposed framework by mapping it with participants’ and institution’s experiences to derive needs (qualitative)
5	Bates [45]	Ghana (LMIC)	Health professionals: medicine, physiotherapy, pharmacy and health management (n = 15)	To evaluate the effectiveness of a 1-year part-time course in research skills	One-year part-time course	2. Multi-study approach: Intervention study in pre-post-test design AND Cross-sectional study	Individual	Outcome evaluation: process and content of course delivery, competencies and confidence	Analysis of students’ research proposals and projects (quantitative); Research Self-Efficacy Scale (quantitative); Analysis of learners’ reflective commentaries (grounded theory approach) (qualitative); Course evaluation (nominal group technique) (qualitative); Pre- and post-test delivery of “Stages Of Change” tool (quantitative);
6	Bates [29]	Ghana (LMIC), Kenya (LMIC), Malawi (LIC) and Democratic Republic of Congo (LIC)	Four case studies with health-related research projects from four different African countries	To develop indicators to monitor the building of sustainable health research capacities	Not described further	1.2.2 Theoretical study	Individual and organizational	Definition of needs: list of capacity gaps, list of critical and supporting stakeholders Outcome evaluation: publications and/or presentations at national/international meetings, expanded skills and workforce, reduction of input of northern partners, long-term funding	Researchers mapped their framework (Bates et al. [21]) with four case studies to derive generalizable indicators (qualitative)
7	Bullock [46]	United Kingdom (HIC)	Healthcare managers from 10 sites within the National Health Service (NHS)^e^	To improve quality of health research by involving healthcare managers in research projects	12-months fulltime programme	1.2.3 Expert study	Individual	Outcome evaluation: motivation, arrangements, experiences, lessons learned and quality improvements of the research and programme	Adapted version of Kirkpatrick’s framework [47, 48] for guiding and coding of semi-structured face-to-face interviews (qualitative);
8	Cooke [49]	United Kingdom (HIC)	General practitioners, nurses, social workers, pharmacists^d^	To find indicators to evaluate the “Designated Research Team” (DRT) approach to build health research capacity in primary and community care settings	Training, mentorship, supervision, partnership development, protected time for research	1.2.2 Theoretical study	Individual/team	Outcome evaluation: constructing and applying indicators	Mapping of Cooke’s framework (Cooke [8]) with a case to derive literature-based and expert-based indicators for evaluating the DRT (qualitative)
9	Corchon [50]	Spain (HIC)	Clinical nurses (n = 170)	To develop nursing research capacity in clinical settings	Mentoring, research courses and journal clubs	1.1.1.2 Intervention study in non-randomized controlled design	Individual	Outcome evaluation: research knowledge, skills, competencies, attitudes, facilitating factors and barriers	Pre- and post-training delivery of Nursing-research-questionnaire (control) (quantitative); Research-knowledge-objective-test (intervention) (quantitative); Facilitators and barriers scale (intervention) (quantitative)
10	Dodani [51]	Pakistan (LMIC)	Health professionals^e^ (n = 56)	To strengthen research capacities through a research skills training workshop in collaboration with the University of Pittsburgh	9-day research training workshop	1.1.2.1 Intervention study in pre-post-test design with 1 year follow-up	Individual	Outcome evaluation: knowledge	Self-constructed 20-item multiple choice questionnaire (quantitative)
11	Du Plessis [52]	Republic of South Africa (UMIC)	Nurses, other health-related researchers, and national and nternational stakeholders^d,e^	To understand the stakeholders’ and nurses’ opinion of meaningful research	Study to prepare any HRCD activity	1.2.3 Expert study	Individual and organizational	Definition of requirements: description of meaningful research	Qualitative secondary analysis with re-exploration of existing data from a Delphi study and focus group discussions
12	Finch [53]	Australia (HIC)	Speech language pathologists (SLP) (n = 158)	To investigate the current research interest, confidence, and experience in the SLP healthcare workforce, and factors that predict research engagement	Study to prepare any HRCD activity	1.2.1 Cross-sectional study	Individual	Analysis of current state: research skills, research participation	Research spider tool and additional questions on research participation (quantitative)
13	Golenko [22]	Australia (HIC)	Allied health senior managers (n = 9)	To describe and analyse allied health senior managers’ perspectives of how organizational factors impact research capacity development	Study to prepare any HRCD activity	1.2.3 Expert study, part of Holden et al. [54]	Organizational	Definition of requirements: organizational factors and support for research-capacity building (RCB), barriers and motivators, research culture	Qualitative study with semi-structured interviews
14	Green [35]	United Kingdom (HIC)	Senior staff with teaching role (nurses and midwifes) (n = 34)	To examine the development of nursing and midwifery research capacity from the faculty perspective	Analysis of institutionalized CD activities	2. Multi-study approach: two expert studies AND Theoretical study	Individual and organizational	Outcome evaluation: research culture, management and organization, problems and challenges, wider context	A case study approach using three types of qualitative methods: Interview; Focus group discussions; Document analysis
15	Henderson-Smart [55]	Australia (HIC), Malaysia (UMIC), Philippines (LMIC), Thailand (UMIC)	Local researchers of four sites from South East Asia^d,e^	To improve the health of mothers and babies in South East Asia by using and generating relevant evidence	Training and support for generating, using and dissemination of evidence	1.1.2.1 Intervention study in pre-post-test design	Individual and organizational	Outcome evaluation: adherence to recommended clinical practices and health outcomes, involvement in evidence-based practice, local barriers	Patient chart analysis if best evidence practice had been followed (qualitative); Survey and document analysis: Involvement in evidence based practice; research activities (mixed); Surveys and interviews: Local barriers to practice change (mixed)
16	Holden [56]	Australia (HIC)	Allied health professionals ^e^ (n = 134)	To develop and validate a questionnaire to evaluate the effectiveness of research culture building activities on individual, team and organizational level	Not described further	1.2.4 Validation study	Individual/team and organizational	Needs and outcome evaluation	The research capacity and culture tool (RCC) (quantitative)
17	Holden [54]	Australia (HIC)	Multidisciplinary primary healthcare teams^d,e^ (8 teams)	To evaluate the effectiveness of a DRT approach to build research capacities using RCC	Supporting teams to conduct small research projects with a multi-strategic approach	1.1.1.2 Intervention study in non-randomized matched-pairs design	Individual/team, and organizational	Outcome evaluation: individual, team and organizational domain	RCC (intervention and control) (quantitative); Qualitative data on contextual information (intervention and control); Qualitative data on team related aspects (intervention)
18	Hyder [32]	Pakistan (LMIC)	Local researchers^e^ (n = 54)	To evaluate the current state and impact of human resource development for health research at doctoral level	Training on health research skills	1.2.1 Cross-sectional study	Individual	Outcome evaluation: training programme characteristics, contributions through research, publications Impact evaluation: teaching activities after returning to Pakistan	Self-constructed questionnaire (quantitative)
19	Hyder [57]	Sub-Saharan Africa	Selected trainees from Sub-Saharan Africa^e^ (n = 12)	To assess given outputs of “The Johns Hopkins-Fogarty African Bioethics Training Programme” (FABTP)	Courses on bioethics, research ethics and research methodology	1.2.1 Cross-sectional study	Individual	Outcome evaluation: enhanced knowledge, new skills, publications, research grants, number of students taught	FABTP evaluation framework: Informal progress notes and evaluation forms (mixed); Transcripts from trainees’ coursework (qualitative); Resumes (qualitative); Formal progress notes (qualitative)
20	Jamerson [30]	United States of America (HIC)	Undergraduate, masters and doctoral nursing students (n = 30)	To describe a training on nursing research capacities	Collaboration between nursing students and clinician researchers	Not mentioned	Individual	Outcome evaluation is unclear	Evaluation design, methods and tools are not described
21	Janssen [36]	New Zealand (HIC)	Physical therapists and clinical managers (n = 25)	To explore the experiences of physical therapists and clinical managers conducting research facilitated by Participatory-Action-Research (PAR) approach	Supporting physical therapists and clinical managers in initiating and conducting research by PAR approach	Multi-study approach: Intervention study in pre-post-test design and 1 year follow-up AND Theoretical study 1.2.3 Expert study	Individual and organizational	Outcome evaluation: experiences related to the initiated research process, motivation, research confidence and orientation	Semi-structured interviews at the end of the intervention and 1 year later (qualitative); Field notes (qualitative); Reflections of PAR groups (qualitative); Three questionnaires in pre-post-test design with 1 year follow-up (quantitative): Edmonton Research Orientation Survey, two visual analogue scales
22	Jones [58]	Australia (HIC)	General practitioners (n = 11)	To determine research training needs and barriers	Study to prepare any HRCD activity	1.2.3 Expert study	Individual and organizational	Analysis of current state: experiences with research, level of research skills, perceived barriers	Grounded theory approach: Semi-structured face-to-face or telephone interviews (qualitative)
23	Kwon [59]	United States of America (HIC)	Community-based organizations (CBO) and partners (n = 27)	To assess the resources and needs for research capacities of CBOs	Study to prepare any HRCD activity	1.2.1 Cross-sectional study	Organizational	Definition of needs: organizational characteristics, involvement in research, research related training, infrastructure	Face-to-face group discussions (qualitative); Online questionnaires (quantitative)
24	Lazzarini [60]	Australia (HIC)	Podiatrists (n = 70)	To report the research capacity of podiatrists	Study to prepare any HRCD activity	1.2.1 Cross-sectional study (part of a longitudinal observational study)	Individual/team and organizational	Analysis of current state: individual research skills, team and organizational aspects of research	Electronic survey (quantitative); RCC tool (quantitative)
25	Levine [24]	United States of America (HIC)	Principal investigators of two research programmes (n = 15)	To evaluate two healthcare research capacity development programmes and their sustainability	Two capacity development programmes on health research infrastructure	1.1.2.1 Intervention study in pre-post-test design with 6 years follow-up	Organizational	Analysis of current state: level of research activities Outcome evaluation: research infrastructure strategies, project barriers and facilitators, process variables, success variables	Mixed-method approach guided by a self-constructed framework: Interviews (qualitative); Secondary sources like annual reports or grant applications, etc. (quantitative); Surveys (quantitative)
26	Mahamood [25]	Bangladesh (LMIC)	Managers, key researchers and external partners^d^	To assess structural and organizational aspects of research capacity development activities	On-going research activities and capacity development strategies	1.2.1 Cross-sectional study	Organizational	Outcome evaluation: perceived problems and issues, structural and organizational performance indicators, financial indicators	Mixed-method approach to re-assess defined issues (guided by a self-constructed framework): Interviews (qualitative); Questionnaires (quantitative); Financial analysis (quantitative); Structural analysis of investigated institution (qualitative)
27	Mayhew [28]	Republic of South Africa (UMIC) and Thailand (UMIC)	Programme staff (n = 25) from two partners in South Africa and one in Thailand^e^	To strengthen health economics-related research capacity through partnerships	North-southern partnerships in research, teaching and communication of new knowledge	Multi-study approach: Theoretical study AND Expert study	Individual/team, organizational and partnerships	Outcome evaluation: characteristics of participants, publications, projects initiated, effects from partnerships	Mixed-method approach guided by evaluation framework: In-depth interviews (qualitative); Document analysis (qualitative); Annual reports and other programme reports (quantitative)
28	McIntyre [61]	Australia (HIC)	Different health practitioners^e^ (n = 105)	To build research capacity and to increase the number of health practitioners with knowledge and skills in health research	Researcher development programme	1.2.1 Cross-sectional study	Individual	Outcome evaluation: knowledge, attitudes and practice in relation to research	Measuring the impact of the training by applying an online-questionnaire (quantitative)
29	Minja [62]	Various developing countries^e^	Participants (n = 128) and institutions (n = 20) of three different capacity development grants^e^	To identify factors that positively influenced and improved the research capacity and career development of grant recipients	30 years training in tropical disease	1.1.2.1 Intervention study: Pre-post-test design study	Individual and organizational	Outcome evaluation: indicators on individual career development, research skills and productivity, indicators on institutional infrastructure and development	Mixed-method approach: three standardized questionnaires for individuals (quantitative); In-depth interviews (qualitative); Questionnaires for institutions (quantitative)
30	Moore [63]	United Kingdom (HIC)	Nurses, midwives, and managing staff within NHS foundation trust (n = 16)	To develop infrastructure for research capacity development	Study to prepare any HRCD activity	1.2.3 Expert study	Organizational	Analysis of current state: barriers and facilitators of the research process	Observing researchers in their natural field by applying the “Action research strategy”: Semi-structured individual interviews (qualitative)
31	Njie-Carr [27]	Uganda (LIC)	Clinicians, community health workers, and administrative staff (n = 43)	To evaluate a research capacity development programme (preparing for the implementation and evaluation of a mobile phone based healthcare training on HIV/AIDS)	Training to conduct and evaluate a mobile-phone-based healthcare programme	1.1.2.1 Intervention study in pre-post-test design	Individual/team and organizational	Definition of needs: pre-training assessment Outcome evaluation: structural and organizational aspects of trainings, research knowledge, skills and confidence	Cooke’s evaluation framework (Cooke [8]): three questionnaires were constructed and delivered at three time points (quantitative): Situational analysis: Pre-training assessment; Interim evaluation of RCB activities; Final or post-training evaluation of RCB activities
32	Otiniano [64]	United States of America (HIC)	Community health workers in Latino communities (n = 8)	To present case studies of eight health promoters who participated in a health policy research programme	3-days course on research terminology and methods and a workshop conducted by the course participants to train their colleagues	1.2.1 Intervention study in pre-post-test design	Individual	Analysis of current state: experiences with data and milestone tracking Outcome evaluation: extent to which new skills were developed	Pre-training assessment: analysis of an application survey (quantitative); Milestone tracking for peer teaching workshops in health research (quantitative); Post-training assessment: qualitative phone interviews guided by the “Grounded Theory” method (qualitative)
33	Pager [65]	Australia (HIC)	Allied health professionals^e^ (n = 84)	To gain a better understanding of how motivators, enablers, and barriers impact research activities within allied health professions	Study to prepare any HRCD activity	1.2.1 Cross-sectional study	Individual/team, and organizational	Analysis of current state: research motivators, enablers and barriers	Written version of research capacity and culture (RCC) tool (quantitative); Tool is broadened to questions on motivators, enablers and barriers on individual and team level (quantitative)
34	Perry [66]	United Kingdom (HIC)	Participants (nurses, midwives, and allied health professionals) and managers (n = 98)	To evaluate the extent to which a research facilitator can provide and improve research skills	Programme on research development, knowledge and implementation	Multi-study approach: Intervention study in pre-post-test design AND Expert study	Individual	Outcome evaluation: processes and activities (participants) and impact of the training (managers)	Mixed-method approach guided by a self-constructed framework: Questionnaire on opinions und perceptions of participants: comparison with previously defined objectives (quantitative); Semi-structured interviews with managers (qualitative)
35	Priest [67]	United Kingdom (HIC)	Nurses, social scientists^d^	To evaluate nursing lecturers’ research capacity by involving them as co-researchers in a research project (for details of this project cf. Green et al. [35, 68] and Segrott et al., [69])	Programme to integrate neophyte researchers in a research project with experienced researchers	1.2.1 Cross-sectional study	Individual	Outcome evaluation: reasons for becoming a member of the study, experiences in terms of benefits and problems	Questionnaire with open-ended questions (mixed); Comparison of these findings with the findings of the main study (Green et al. [35, 68], Segrott et al. [69]) (quantitative)
36	Redman-Maclaren [70]	Australia (HIC) and Solomon Islands (LMIC)	Solomon Islander and Australian researchers^e^ (n = 10)	To explore the benefits of a collaborative research capacity development strategy for both Australian and Solomon Islander researchers	Two-week workshop on research design, data collection and reporting with teaching strategies	1.2.3 Expert study	Individual and organizational	Outcome evaluation: benefits, barriers, experiences, future development	Grounded theory method was applied: four open ended questions either in a face-to-face interview or in written form (qualitative)
37	Ried [71]	Australia (HIC)	Primary healthcare professionals^e^ (n = 89)	To develop and assess research and evaluation skills among primary healthcare professionals	Study to prepare any HRCD activity	1.2.1 Cross-sectional study	Individual	Analysis of current state and definition of needs: current level of participation in research, level of experience in 10 specific research skills, publication and funding record, interest in training, etc.	Questionnaire with five topics; Visual research spider tool (part of the questionnaire) (quantitative)
38	Salway [72]	United Kingdom (HIC)	Public health staff (n = 10)	To evaluate and identify elements of learning of participants within a certain research capacity development programme	5-month research capacity development programme	1.2.1 Cross-sectional study	Individual	Outcome evaluation: participants perception of learning, experiences, programme content and programme structure	Post workshop evaluation forms (quantitative); Final evaluation with structured and open ended questions (mixed); Follow-up evaluation 12 months later with three open ended questions (mixed)
39	Suter [31]	Canada (HIC)	13 case reports	To describe the process used by the Community of Practice to initiate research capacity development	Study to prepare any HRCD activity	1.2.2 Theoretical study	Individual and organizational	Definition of requirements: research and evaluation skills, support of research and evaluation, building linkages, ensuring dissemination, building sustainability, creating appropriate infrastructure	Mapping recommendations of 13 case reports against Cooke’s framework (Cooke [8]) (qualitative)
40	Webster [73]	Australia (HIC)	Health professionals^e^, managers and mentors (n = 25)	To gain better understanding of the impacts of research programme from the participants’, managers’, and mentors’ perspectives	2-years health research capacity development programme	1.2.3 Expert study	Organizational	Outcome evaluation: effectiveness of the partnership, leadership, workforce development, resource allocation and organizational change strategies	Semi-structured interviews (qualitative)
41	Wilson [74]	Sites outside the United States of America^e^	Clinical research managers^e^ (n = 166)	To describe the development, implementation, and evaluation of a distance-based continuing education programme for study coordinators outside of the United States of America	2-years online programme on clinical research	1.1.2.1 Intervention study in pre-post-test design	Individual	Outcome evaluation: participants perceptions on the course and teaching strategies, level of knowledge, logs on participants capacity development activities	Modified standard course, teaching and overall programme evaluation forms from the University of Alabama (quantitative); 21-item investigator-developed online survey to assess students’ level of knowledge at pre and post course time 10-item survey for withdrawals were constructed (quantitative)
42	Wootton [75]	Two countries^e^	Researchers^e^ (n = 82)	To generate a useful “research output score” out of three indicators to measure individual research output	Not described further	1.2.4 Validation study	Individual	Outcome evaluation: development and testing of the “research output score”	Definition of three indicators, which build the “research output score”: grant income, publication and number of PhD students supervised; Application of indicators/research output score in different research departments/countries (quantitative)

^a^Country group by income according to the World Bank: HIC, High-income country; UMIC, Upper-middle-income country; LMIC, Lower-middle-income country; LIC, Low-income country.

^b^Sample size.

^c^See also Figure 2.

^d^Sample size not specified.

^e^Not specified in the article.

Around half of the NaME studies on HRCD activities were conducted in high-income countries (n = 24) [26]. Six studies took place in lower-middle-income and two in upper-middle-income economies. Participants of one study were from a low-income country [27]. Two studies were performed in partnerships between a high-income and several low-, lower-middle and upper-middle-income economies. Mayhew et al. [28] described a partnership study between two upper-middle income countries and Bates et al. [29] analysed case studies from two lower-middle-income and two low-income economies. Five authors did not specify the country or region of their studies.

The evaluation focus of the studies was predominately on outcome evaluation (n = 23). Besides that, six studies surveyed the current state, three studies assessed requirements, and two studies investigated needs of HRCD activities. The remaining eight studies combined two evaluation aspects: definition of needs and outcome evaluation (n = 4), analysis of current state and outcome evaluation (n = 1), outcome evaluation and impact evaluation (n = 1), and analysis of current state and definition of needs (n = 1). Jamerson et al. [30] did not define their focus of evaluation.

Nearly half of the studies investigated HRCD on the individual/team level (n = 20); 16 studies were conducted at both the individual/team and organizational levels. The authors of six studies focused on organizational aspects of HRCD.

Almost all studies (n = 38) described and evaluated HRCD activities; 19 of these HRCD activities were training programmes of predefined duration, lasting between some hours or days up to 2 years. Another nine HRCD activities were perpetual or their duration not specified and 10 studies defined and pre-assessed the setting in preparation of an HRCD activity. The authors of four studies did not specify an HRCD activity, focussing on the development or validation of tools, instruments, and frameworks.

The participants of HRCD activities represent a wide range of health professions (e.g. laboratory scientists, physiotherapists, dentists, pharmacists); 10 studies investigated staff with management tasks in health, e.g. hospital managers, clinical research managers. Nurses participated in eight studies with another eight studies looking into ‘research staff’ and ‘scientists’ with no further description. Medical practitioners were studied in five papers. Besides all these, the background of participants was often not specified beyond general terms like ‘health professionals’, ‘ethic committee members’, ‘scholars’, ‘university faculty members’, or ‘allied health professionals’. In a different approach, Suter et al. [31] analysed reports and Bates et al. [29] investigated case studies (without specifying the material scrutinized).

A wide variety of study designs was employed by the studies included in the review. We identified 35 single-study and six multi-study approaches. Of the 35 single-study approaches, 10 were designed as intervention (three with control groups) and 25 as non-intervention studies. Four multi-study approaches combined an intervention study with a non-intervention study. Two multi-study approaches combined different non-intervention studies. Jamerson et al. [30] did not specify their study design.

Many different tools and instruments for NaME were identified and applied in quantitative, qualitative and mixed mode of analysis. No preferred approach was observed. One third of the studies (n = 16) used a combination of tools for quantitative as well as qualitative analysis. In 13 studies, tools like questionnaires and assessment sheets were applied to evaluate and monitor HRCD activities quantitatively. Evaluation tools, such as interviews, focus group discussions, document analyses, or mapping of cases against evaluation frameworks, were identified in 12 studies and commonly analysed in a qualitative approach. In one study, tools for evaluation were not described at all.

## Discussion

### Summary of evidence

The aim of our systematic review was to give an overview on tools and instruments for NaME of HRCD activities on the individual and organizational level; 42 included articles demonstrated a large variety of tools and instruments in specific settings. Questionnaires, assessment sheets and interviews (in qualitative settings) were most commonly applied and in part disseminated for further use, development and validation.

Overall, 36 studies were either conducted on the individual/team or on both individual/team and organizational level. Within these studies, a well-balanced mixture of quantitative, qualitative and mixed tools and modes of analysis were applied. Judging from the depth of these studies, it seems as if NaME of HRCD on the individual level is quite well developed. Only six studies focused exclusively on organizational aspects, almost all with qualitative approaches, indicating that HRCD studies at this level are still mainly exploratory. The organizational level is possibly a more complex construct to measure. The fact that 13 out of 19 studies that broach organizational aspects were conducted in high-income countries might reflect the wider possibilities of these research institutions and indicates a need for more attention to NaME on the organizational level in lower-income settings. Results from these exploratory studies on the organizational level should feed into the development of standardized quantitative indicators more regularly. Qualitative approaches could be pursued for complex and specific constructs not easily covered quantitatively.

By not limiting the primary selection of articles for this review to a specific health profession, it was revealed that staff with management tasks in health research, as well as nurses, were the cohorts most frequently targeted by NaME studies. Further research should concentrate on other health professionals to determine communalities and differences of health-research related skill acquisition and development between health professions. These studies could determine whether and which parts of HRCD and NaME can be considered generic across health professions. Further, we will at some point have to ask, who is being left out and who is not getting access to HRCD programs, and why.

The focus of NaME throughout the studies included in this review was on outcome measurement, regardless of whether these were conducted in high-income, upper-middle, lower-middle, or low-income countries. However, there were only few reports of needs assessment from middle- and low-income economies, while high-income countries regularly give account of current states. While this should not be over-interpreted, it still raises the question of whether the needs assessment in the middle- and low-income countries is being done as thoroughly as warranted, but not reported in the articles, or if these countries’ needs might not always be at the very centre of the HRCD’s attention. While the evaluation of HRCD outcomes is, of course, of importance, more attention should be paid to the sustainability of programs and impact evaluation, e.g. parameters of patient care or societal aspects. Only one study, that of Hyder et al. [32], made use of one such indicator and assessed the impact of a HRCD training by considering “teaching activities after returning to Pakistan”. The development of valid impact indicators of course constitutes a methodological challenge. Some studies reporting impact evaluation on a system level might of course have been missed due to the search parameters applied.

When undertaking the review, three main methodological weaknesses of this research area became apparent. First, there is a need for common definitions and terminologies to better communicate and compare the HRCD efforts. The analysis of the studies showed that there is an inconsistent use of terms, for example, for CD activities (e.g. training, course, or workshop). Similar problems were already identified in the context of educational capacity building by Steinert et al. [33], who suggest definitions for different training settings which may also be suitable for a more precise description of CD activities. A common taxonomy for the description of health professionals (i.e. the study participants) would be just as desirable. The use of coherent terms would not only enable the accurate replication of studies but also help in determining whether tools and instruments from one setting can be easily transferred to another. A clear and coherent description of study setting and participants is thus an integral step towards scientific transparency. The incoherent categorisation of study types is probably not a new problem. It is, however, amplified by authors who choose very complex approaches to collect data at different NaME levels with deviating terms to describe these approaches [28, 34–36].

The second weakness of the research area is the varying adherence to reporting standards. While there are standards available for reporting qualitative or quantitative research (e.g. Rossi et al. [12], Downing [37], Mays & Pope [38]), it seems these or similar recommendations were not frequently considered when reporting or reviewing NaME studies. This was particularly the case in studies with a mixed-method mode of analysis, where the need for more standardised reporting became apparent. Frambach et al.’s [39] “Quality Criteria in Qualitative and Quantitative Research” could provide guidance, especially for studies with mixed-method approaches. Another important aspect of transparent reporting would be the publication of the tools and instruments used in NaME studies. Of the 42 articles scrutinized during this review, only 15 either disclosed the tools and instruments within the article itself in an appendix or volunteered to have them sent to any audience interested. Of all the tools and instruments disclosed, only two were used in two or more studies. Making the tools and instruments available to the HRCD community would not only allow for their adaptation whenever necessary but, more importantly, support their validation and enhancement.

The last point concerns the study designs implemented. The majority of articles are mainly descriptive, non-intervention studies that only allow for low evidence according to Cochrane standards [40]. While most HRCD studies conducted in high-income economies were of non-interventional nature, those from low- and middle-income countries were a mix of non-intervention, intervention and multi-study approaches, yielding higher levels of evidence. Of all interventional studies, most employed a quasi-experimental design with only one randomized controlled trial [23]. The studies reporting HRCD on the institutional level were also primarily on a descriptive level. Cook et al. [41], however, demand going beyond describing what one did (descriptive studies) or whether an intervention worked or not (justification studies). Instead, they call for analysing how and why a program worked or failed (clarification studies). An in-depth analysis of the effectiveness of different HRCD activities is, however, still lacking.

### Limitations of the systematic review

This systematic review displays some methodological limitations itself. The issue of deviating terminologies has been raised earlier. In most cases, we adopted the terms used in the studies themselves, e.g. when reporting the authors’ denoted study designs. In very few cases, we changed or completed terms to make the studies more comparable to others. One example is changing the wording from Green et al.’s [35] “case study approach” into a “multi-study approach” to match Flyvberg’s taxonomy [42]. Other limitations typical for reviews may also apply. Relevant sources might not have been detected due to the selected search terms, the range of the data sources, the exclusion of grey literature, and the restriction to English and German sources.

## Conclusion

A systematic review on studies from the field of HRCD activities was conducted, with 42 studies being fully analysed. The analysis revealed that a variety of terms and definitions used to describe NaME efforts impedes the comparability and transferability of results. Nevertheless, insight from this review can help to inform researchers and other stakeholders in the HRCD community. A coherent overview on tools and instruments for NaME of HRCD was developed and is provided (Table 3).

Furthermore, it is time to set standards for NaME in the HRCD community. Researchers and stakeholders should develop a common research agenda to push, systematise and improve the research efforts in the field of NaME of HRCD activities. To do so, a common language and terminology is required. The conceptualizations used for the purpose of these review can inform this development. On the other hand, we have to critically analyse research gaps in terms of generalizable versus context-specific theories, methods, tools, and instruments. To maximize the benefits and to incorporate different research traditions, these undertakings should be done internationally and multi-professionally within the HRCD community.
